# Correction: Wilk et al. “The Effects of High Doses of Caffeine on Maximal Strength and Muscular Endurance in Athletes Habituated to Caffeine” Nutrients, 2019, 11(8), 1912

**DOI:** 10.3390/nu11112660

**Published:** 2019-11-04

**Authors:** Michal Wilk, Michal Krzysztofik, Aleksandra Filip, Adam Zajac, Juan Del Coso

**Affiliations:** 1 Institute of Sport Sciences, Jerzy Kukuczka Academy of Physical Education, 40-065 Katowice, Poland; m.krzysztofik@awf.katowice.pl (M.K.); a.filip@awf.katowice.pl (A.F.); a.zajac@awf.katowice.pl (A.Z.); 2 Exercise Physiology Laboratory, Camilo José Cela University, 28692 Madrid, Spain; jdelcoso@ucjc.edu

The authors wish to make a correction to the published version of their paper [1]. We noticed an error in the statistical analysis that requires correcting, as it may contribute to an incorrect understanding of our study’s scientific results and conclusions. In the study Section 2.7 (Statistical Analysis), the identification of differences between the placebo (PLAC) and the two doses of caffeine under experimentation 9 and 11 mg/kg/b.m.; CAF-9 and CAF-11, respectively) was performed using a one-way ANOVA. As the 16 participants of this investigation underwent all the experimental trials and acted as the own controls, the correct statically approach should have included the use of a one-way repeated measures ANOVA. After running this new statistical analysis, some new differences have appeared between PLAC and the use of caffeine, in addition to the new *p* values for each comparison. The repeated measures ANOVA revealed statistically significant differences in 1RM (*p* < 0.01), MP (*p* < 0.01) and PP (*p* = 0.04) between PLAC vs. CAF-9 and CAF-11, in addition to the difference in PV (*p* < 0.01) that was already presented in the previous version of the manuscript (Table 1). Tukey’s post-hoc tests revealed a significantly higher 1RM in CAF-9 and CAF-11 trials when compared to PLAC and significantly lower MP, PP and PV in the CAF-11 trial when compared to PLAC (Table 2).

Although the original experimental results remain unchanged, this new and correct statistical analysis indicates that the acute intake of high doses of CAF (9 and 11 mg/kg/b.m.) was effective to produce statistically measurable ergogenic effect on the bench press 1RM in individuals habituated to CAF intake. In case of muscular endurance, the intake of 11 mg/kg/b.m. significantly decreased MP, PP and PV during bench press testing performed to concentric muscle failure in these habitual caffeine users. 

The authors apologize to the readers for any inconvenience caused by this modification. The original manuscript will remain online on the article webpage with a reference to this correction.

## Figures and Tables

**Table 1:** Summary of performance data under the three employed conditions.

Variable	Placebo (95% CI)	CAF-9 (95% CI)	CAF-11 (95% CI)	F	*p*
1RM [kg]	118.3 ± 14.5 (109.4 − 125.5)	122.3 ± 15.3 (115.7 − 132.5)	124.2 ± 11.4 (116.3 − 135.2)	7.46	0.01 *
T-REP [n]	25.1 ± 3.2 (23.3 − 26.8)	25.0 ± 4.9 (22.4 − 27.6)	25.6 ± 3.3 (23.8 − 27.3)	0.14	0.86
TUT_CON_ [s]	17.1 ± 3.29 (15.3 − 18.8)	19.1 ± 3.29 (17.3 − 20.8)	16.9 ± 3.39 (15.1 − 18.8)	2.67	0.08
MP [W]	348 ± 79 (305 − 390)	333 ± 72 (294 − 372)	318 ± 78 (276 − 360)	6.07	0.01 *
PP [W]	798 ± 164 (710 − 886)	766 ± 134 (694 − 837)	731 ± 186 (632 − 831)	3.27	0.04 *
MV [m/s]	0.71 ± 0.10 (0.66 − 0.76)	0.67 ± 0.08 (0.63 − 0.72)	0.70 ± 0.07 (0.66 − 0.74)	1.39	0.26
PV [m/s]	1.39 ± 0.16 (1.31 − 1.48)	1.37 ± 0.15 (1.29 − 1.45)	1.25 ± 0.17 (1.16 − 1.34)	6.09	0.01 *

All data are presented as mean ± standard deviation; CI—Confidence interval; * statistically significant difference *p* < 0.05; 1RM: One repetition maximum; T-REP: Total number of repetitions; TUT_CON_: Time under tension during concentric movement; MP: Mean power output; PP: Peak power output; MV: Mean velocity; PV: Peak velocity.

**Table 2:** Differences in placebo vs. caffeine conditions between experimental trials.

Variable	Comparison	*p*	Effect Size (Cohen *d*)	Relative Effects [%]
1RM [kg]	Placebo vs. CAF-9	0.01 *	0.26—small	3.3 ± 4.1
	Placebo vs. CAF-11	0.01 *	0.45—small	4.7 ± 5.1
T-REP [n]	Placebo vs. CAF-9	0.99	−0.02—negative effects	0.4 ± 12.1
	Placebo vs. CAF-11	0.90	0.15—small	2.0 ± 11.2
TUT_CON_ [s]	Placebo vs. CAF-9	0.14	0.6—moderate	10.5 ± 15.5
	Placebo vs. CAF-11	0.99	−0.05—negative effects	−6.2 ± 21.5
MP [W]	Placebo vs. CAF-9	0.21	−0.19—negative effects	−1.5 ± 7.6
	Placebo vs. CAF-11	0.01 *	−0.38—negative effects	−9.4 ± 10.5
PP [W]	Placebo vs. CAF-9	0.44	−0.21—negative effects	−4.2 ± 8.3
	Placebo vs. CAF-11	0.04 *	−0.38—negative effects	−9.2 ± 11.6
MV [m/s]	Placebo vs. CAF-9	0.25	−0.44—negative effects	−6.0 ± 11.8
	Placebo vs. CAF-11	0.86	−0.11—negative effects	−1.4 ± 6.6
PV [m/s]	Placebo vs. CAF-9	0.84	−0.12—negative effects	−1.5 ± 10.2
	Placebo vs. CAF-11	0.01 *	−0.84—negative effects	−11.2 ± 10.7

All data are presented as mean ± standard deviation; * statistically significant difference *p* < 0.05; 1RM: One repetition maximum; T-REP: Total number of repetitions; TUT_CON_: Time under tension during concentric movement; MP: Mean power output; PP: Peak power output; MV: Mean velocity; PV: Peak velocity.
